# Preferential Recruitment of Th17 Cells to Cervical Cancer via CCR6-CCL20 Pathway

**DOI:** 10.1371/journal.pone.0120855

**Published:** 2015-03-13

**Authors:** Qing Yu, Xiang-ming Lou, Yan He

**Affiliations:** 1 Department of Obstetrics and Gynecology, Hangzhou Obstetrics and Gynecology Hospital, Hangzhou First People’s Hospital, Hangzhou, China; 2 Department of Pathology, Yiwu Maternity and Child Care Hospital, Yiwu, Zhejiang Province, China; University of Navarra, SPAIN

## Abstract

Our previous studies suggest that Th17 cells accumulate within tumor tissues and correlate with recurrence of cervical cancer patients. However, the source of the increased tumor-infiltrating Th17 cells remains poorly understood. We investigated the prevalence, phenotype and trafficking property of Th17 cells in patients with cervical cancer. Our results showed that Th17 cells highly aggregated within tumor tissues in an activated phenotype with markedly increased expression of CCR6. Correspondingly, level of CCL20 in the tumor tissues was significantly higher than that in non-tumor and normal control tissues, and strongly positively associated with Th17 cells. Further, in vitro migration assay showed CCL20 had effective chemotaxis to circulating Th17 cells. In conclusion, Th17 cells are recruited into tumor tissues preferentially through CCR6-CCL20 pathway, which can serve as a novel therapeutic target for cervical cancer.

## Introduction

Cervical cancer is the second most common cancer in women worldwide[1]. There is growing evidence that CD4^+^ T-helper (Th) cells play an important role in maintaining immune responses against cancer [2, 3]. Th17 cells are a novel subset of interleukin IL-17-producing CD4^+^ T cells [4] that have been shown to play a crucial role in inflammation and autoimmune disease[5–7], while also accumulating in tumors such as hepatocellular carcinoma[8], gastric cancer[9], lung cancer[10], and esophageal carcinoma[11]. Our previous study also found increased number of Th17 cells in tumor tissues of cervical cancer and their independent association with recurrence after operation[12]. These studies suggest that Th17 cells may contribute to the immunopathogenesis of many types of cancers.

Before T cells can exert their effects on cancer cell, they have to reach target site. The migration of T cells to the target site is a multi-step procedure, in which signals from chemokines/chemokine receptors play a critical role [13]. Different CD4^+^ T cell populations in humans and mice display distinct patterns of chemokine receptor expression. In tumor microenvironment, Th1 cells express CCR5 and CXCR1, Th2 cells express CCR4 and CCR8, whereas regulatory T cells mainly express CCR4[14]. Recent studies suggest that Th17 cells express CCR2, CCR4, and CCR6 in adult human’s peripheral blood[15, 16]. However, the migratory determinants for Th17-cell migration into tumor tissues of cervical cancer patients remain unknown. In this study, we found that the CCR6-CCL20 axis mainly determines the migration of circulating Th17 cells into tumor tissues in cervical cancer patients.

## Patients and Methods

### Ethics Statement

The study protocol was approved by the Institutional Review Board of Hangzhou Obstetrics and Gynecology Hospital. Informed written consent was obtained from patients according to the Declaration of Helsinki.

### Subjects

A total of 35 patients with cervical cancer, who underwent surgical resection at the First People’s Hospital of Hangzhou between 2012 and 2013, were enrolled in this study. Matched peripheral blood (PB), tumors, and corresponding non-tumor tissues were obtained from these patients. None of the patients received anticancer therapy or other medical interventions. The patient characteristics (as it is noted in S1 Table).

### Isolation of PBMC, TIL, and NIL

Peripheral blood mononuclear cells (PBMC) were isolated by Ficoll-Hypaque (Sigma-Aldrich, St. Louis, MO) density gradient separation. Tumor-infiltrating lymphocytes (TIL) and non-tumor-infiltrating lymphocytes (NIL) were isolated as described by Pang and his colleague [17]. Briefly, the tissue was cut into small pieces and incubated in an enzyme mixture containing 0.05% collagenase IV (Invitrogen, Carlsbad, CA) and 0.001% DNase I (Sigma-Aldrich) for 1h. Dissociated tissues were then ground through a 70-μm strainer, and mononuclear cells were obtained by density gradient separation using Ficoll-Hypaque.

### Flow cytometry

PBMC, TIL and NIL were stained with fluorochrome-conjugated monoclonal antibodies against human CD3, CD4, CD45RO, HLA-DR, CCR2, CCR4, CCR6, CD11a, CD49d, CD103, PD-1, Granzyme B, and IL-17 (BD PharMingen, San Diego, CA). Cells were stimulated to secrete cytokines for 4h with 100 ng/mL phorbol myristate acetate (PMA), 1 μg/mL ionomycin (Sigma—Aldrich) and 2 μM monensin (Enzo, Plymouth, PA). For intracellular staining, the cells were permeabilized and fixed using Cytofix/Cytoperm (BD PharMingen) according to the manufacturer’s instructions. After staining, three- or four-color flow cytometry was performed using LSR II flow cytometer (Becton Dickinson, San Jose, CA), and data were analyzed using Flowjo software (Tree Star, Inc., Ashland, OR).

### Immunohistochemical staining

Paraffin-embedded, formalin-fixed liver tissue was cut into 4-μm sections. Antigen retrieval was preformed via pressure cooking for 10 minutes in citrate buffer (pH 6.0). Antibodies of mouse anti-human FoxP3 (dilution, 1:200; Abcam, Cambridge, UK) and rabbit anti-human IL-17 (dilution, 1:150; R&D System, Minneapolis, MN) were used for the primary antibodies. Diaminobenzidine is used for substrate following counterstaining with hematoxylin for single staining.

### Real-time PCR

RNA was extracted using the RNeasy Mini kit (Qiagen, Hilden, Germany) and synthesized for cDNA using QuantiTech Reverse Transcription kit (Qiagen). Quantitative real-time PCR was conducted in SYBR Green PCR Master Mix (Applied Biosystems, Foster City, CA) using the ABI Prism 7500 Real-time PCR System (Applied Biosystems). The following primers were used: GAPDH: CTC TCT GCT CCT CCT GTT CGA C (forward), TGA GCG ATG TGG CTC GGC T (reverse); CCL17: CCA GGG ATG CCA TCG TTT (forward), GGT GGA GGT CCC AGG TAG TC (reverse); CCL20: GAC ATA GCC CAA GAA CAG AAA (forward), GAC AAG TCC AGT GAG GCA CAA (reverse); and CCL22: GGA GGC AAA GAG TAG GGT GTA AT (forward), TCA GCC AGA AAG GCA TAG ATA GA (reverse). Samples were run in triplicate, and their relative expression was calculated in the following formula using GAPDH as endogenous controls: 2^-ΔΔCt^.

### Chemotaxis assay

Migration assays were performed on PBMC using the Transwell system, as previously described [18]. PBMC (1×10^6^) in a volume of 200μl were added to the upper wells (insert pore size, 5μm; Millipore, Billerica, MA). Human chemokines (CCL20, CCL21 and CCL22, 100 ng/ml of each; all from R&D System, Minneapolis, MN), or supernatant of HeLa cells, Siha cells or C-33A cells (Cell Research Institute of the Chinese Academy of Sciences, Shanghai, China) were added to the lower chamber in a volume of 900μl. The HeLa cells (HPV-18 infected cervical cell lines), Siha cells (HPV-16 infected cervical cell lines) and C-33A cells (HPV negative cervical cancer cells) were cultured in RPMI-1640 medium supplemented with 10% FCS in a humidified 5% CO2 at 37°C. The supernatant of cancer cells used for chemotaxis assay was collected after 3- to 5-day-old culture. Antibodies to CCL20, CCL21 and CCL22 (R&D System) were added just before the chemotactic experiments at a saturated concentration of 500ng/ml. After 4 h of chemotaxis at 37°C, cells in the lower wells were collected, and stimulated with 100 ng/mL PMA and 2 μM monensin for 4h. The cells were then permeabilized and fixed using Cytofix/Cytoperm (BD PharMingen) and stained with fluorochrome-conjugated against IL-17. The absolute number of cells was counted and calculated using the Perfect Count System (Cytognos, Salamanca, Spain). To calculate the migration index, the number of migrated cells for chemokines was divided by the number of migrated cells for the control medium [19].

### Statistic analysis

All statistical data were analyzed using SPSS, version 16.0. Differences between groups were analyzed using one-way ANOVA, Mann-Whitney U test, or χ2 test where appropriate. Pearson coefficient was computed to assess the association between chemokine levels and Th17 cells in the tumor environment. Data were expressed as means ± S.E.M. Statistical significance was set at *P* < 0.05.

## Results

### Distribution of Th17 cells in patients with cervical cancer

**Fig 1:**
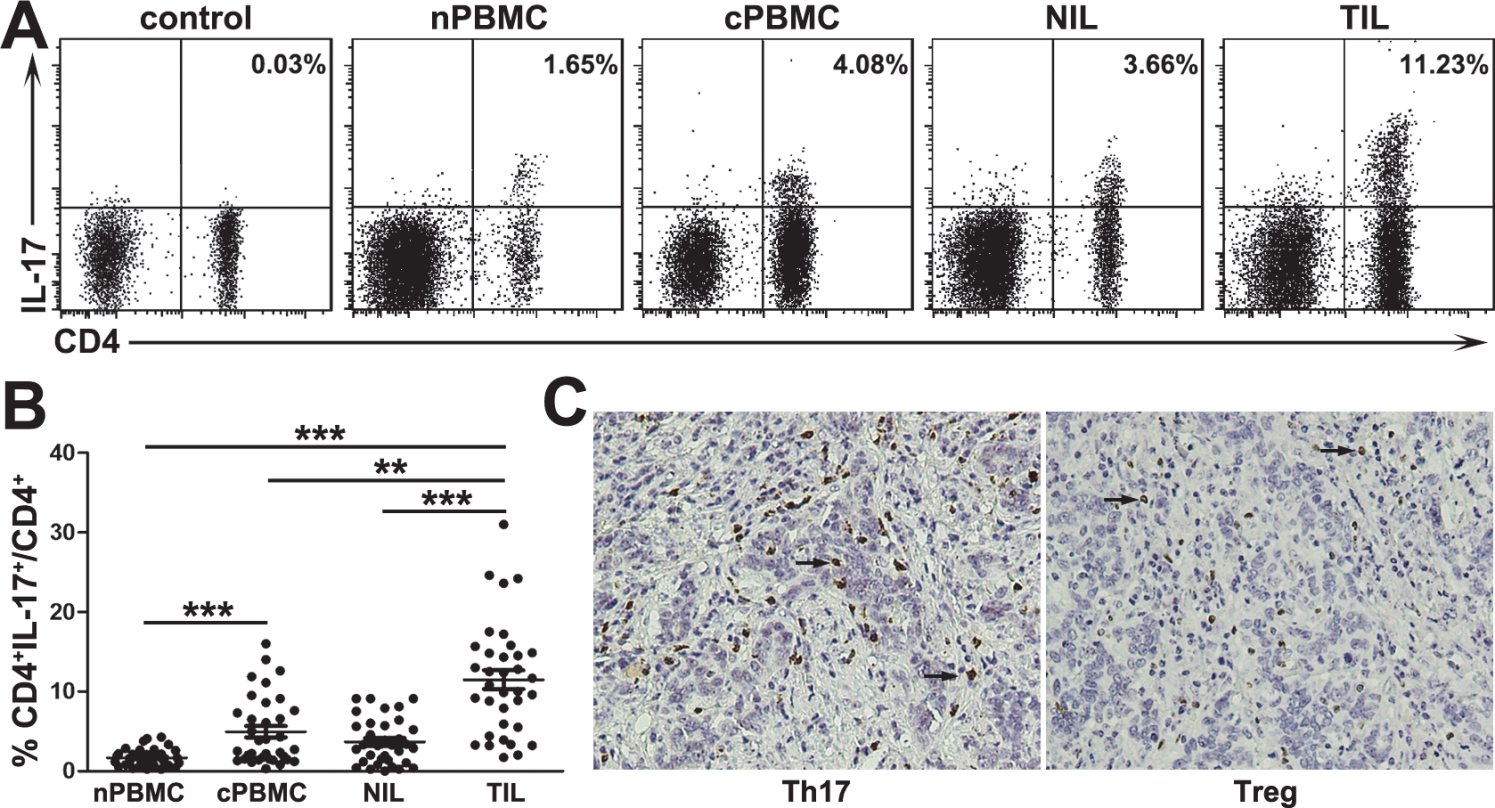
Th17 cells are highly enriched in tumors of patients with cervical cancer. Th17 cells were gated from CD3^+^ T cells by flow cytometry. (A) Representative IL-17 expression profiles in CD4^+^ T cells from the four studied groups. The percentages represent the frequency of Th17 cells among CD4^+^ T cells. (B) Statistical analysis show that the frequency of Th17 cells was higher in patients with cervical cancer, especially among tumor-infiltrating lymphocytes (n = 35). **P < 0.01, ***P < 0.001. (C) Representative images for Th17 cells (IL-17^+^) and Tregs (FoxP3^+^) infiltration in cervical cancer tissue from the same patient. Immunostained cells (brown, indicated by black arrow) and tumor cells (blue). Magnification,×100.

To study the distribution of Th17 cells at tumor site, NIL and TIL were isolated from paired nontumor and tumor tissues in 35 patients with cervical cancer and characterized via flow cytometry (Fig. 1A). As expected, the frequency of Th17 cells in tumor tissue, representing 11.49 ± 1.19% of CD4^+^ T cells, was significantly higher than those in non-tumor tissue (3.68 ± 0.48%, *P* < 0.001), and peripheral blood of patients with cervical cancer (4.96 ± 0.72%, *P* < 0.01) and healthy donors (1.67 ± 0.19%, *P* < 0.001; Fig. 1B). Additionally, the proportion of circulating Th17 cells was also higher in patients with cervical cancer compared with healthy donors (*P* < 0.001). Immunohistochemical staining also observed substantial Th17 cells in tumor tissue of cervical cancer, even higher than regulatory T cells (Tregs) in the same environment (Fig. 1C). These results collectively suggest that Th17 cells are highly enriched in patients with cervical cancer, especially in tumor tissue.

**Fig 2:**
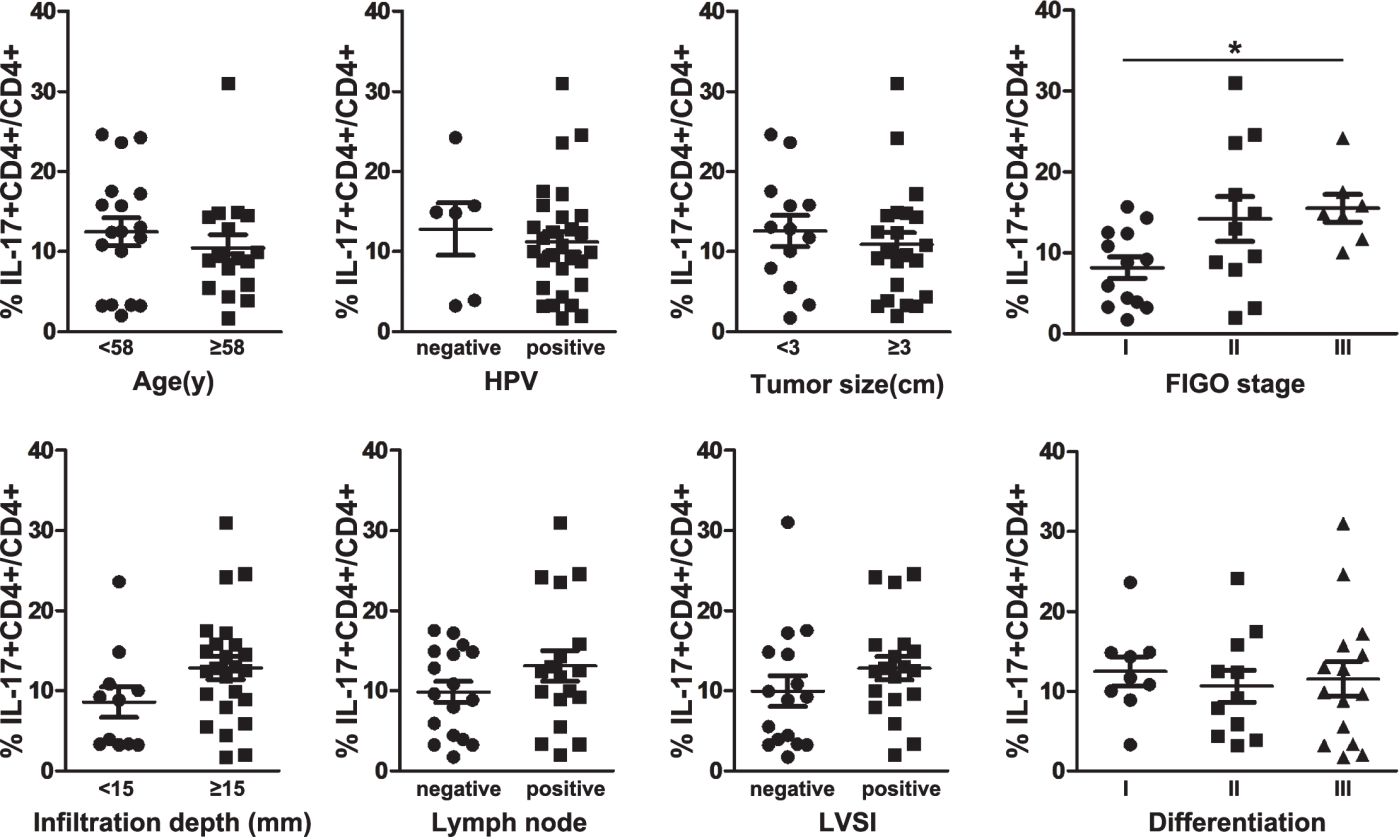
Association of intratumoral Th17-cell prevalence with clinical parameters.

Furthermore, the prevalence of Th17 cells was higher in advanced stage tumors than that in early stage tumors (8.12 ± 1.31% in FIGO I vs. 14.18 ± 2.78% in FIGO II vs. 15.50 ±1.73 in FIGO III; Fig. 2). However, there were no significant differences in the prevalence of Th17 cells for age, HPV status, tumor size, infiltration depth, lymph node status, lymph-vascular space invasion and tumor differentiation (Fig. 2).

Comparison of the frequency of tumor-infiltrating Th17 cells in cervical cancer patients at different age, HPV status, tumor size, FIGO stage, infiltration depth, lymph node status, lymph-vascular space invasion (LVSI) and tumor differentiation. Statistical analysis showed the frequency of Th17 cells in tumor tissues was increased from FIGO I to FIGO III, but not associated with the other clinical parameters. *P < 0.05.

### Phenotypic characteristics of tumor-infiltrating Th17 cells

**Fig 3:**
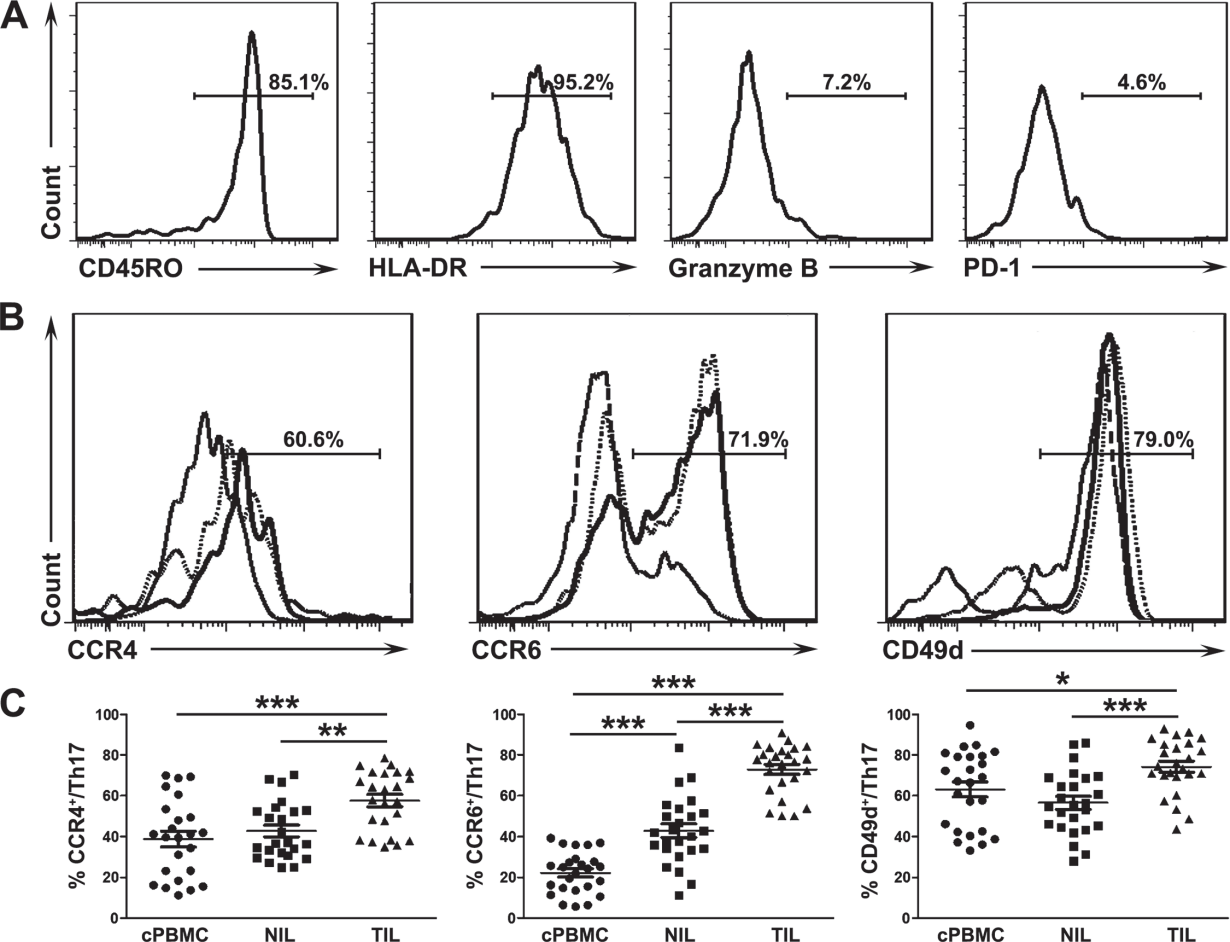
Phenotypic analysis of Th17 cells in patients with cervical cancer. (A) Representative expression profiles of CD45RO, HLA-DR, Granzyme B and PD-1 in tumor-infiltrating Th17 cells. The percentages represent the frequencies of various markers in Th17 cells. (B) Representative expression profiles of CCR4, CCR6 and CD49d on Th17 cells from peripheral blood (long dotted line), non-tumor (dotted line) and tumor tissues (solid line). The percentages represent the frequencies of various markers on tumor-infiltrating Th17 cells. (C) Statistical analysis of surface expression of CCR4, CCR6, CD49d on Th17 cells from peripheral blood, non-tumor and tumor tissues (n = 25). *P < 0.05, **P < 0.01, ***P < 0.001.

We then analyzed the expression of markers related to activation/effector function (CD45RO and HLA-DR) and immune suppression (granzyme B and PD-1). Tumor-infiltrating Th17 cells expressed high CD45RO, HLA-DR, but low granzyme B and PD-1 (Fig. 3A). Granzyme-B-dependent cytolysis[20] and B7-H1/PD-1 pathway[21] contribute to immune suppression in the tumor microenvironment. These results indicated that tumor-infiltrating Th17 cells exhibited an activated memory phenotype (CD45RO^+^HLA-DR^+^) but may not mediate effector function through the granzyme B or B7-H1/PD-1 pathway (Granzyme B^-^/PD-1^-^).

Lymphocyte migration occurs through a multistep process, where chemokine/chemokine receptor signaling is an essential event, followed by integrin-mediated adhesion to vessel walls[13]. Therefore, we further analyzed the surface expressions of chemokine receptors (CCR2, CCR4 and CCR6) and integrins (CD49d, CD11a and CD103) on Th17 cells. Tumor-infiltrating Th17 cells expressed high levels of CCR4 (cPBMC: 38.83 ± 3.90%, NIL: 42.76 ± 2.84%, TIL: 57.61 ± 2.99%; *P* < 0.001 and < 0.01 for TIL vs. cPBMC and vs. NIL, respectively), CCR6 (cPBMC: 22.17 ± 2.04%, NIL: 42.89 ± 3.23%, TIL: 72.97 ± 2.45%; *P* < 0.001 for TIL vs. cPBMC and vs. NIL), and CD49d (cPBMC: 63.13 ± 3.74%, NIL: 56.59 ± 3.10%, TIL: 74.24 ± 2.69%; *P* < 0.05 and < 0.001 for TIL vs. cPBMC and vs. NIL, respectively) (Fig. 3B and 3C), but not CCR2, CD103, and CD11a (data not shown). The expressed homing molecules may be associated with Th17-cell migration and retention within tumor[22].

### Associations of chemokine levels with intratumoral Th17 cells

**Fig 4:**
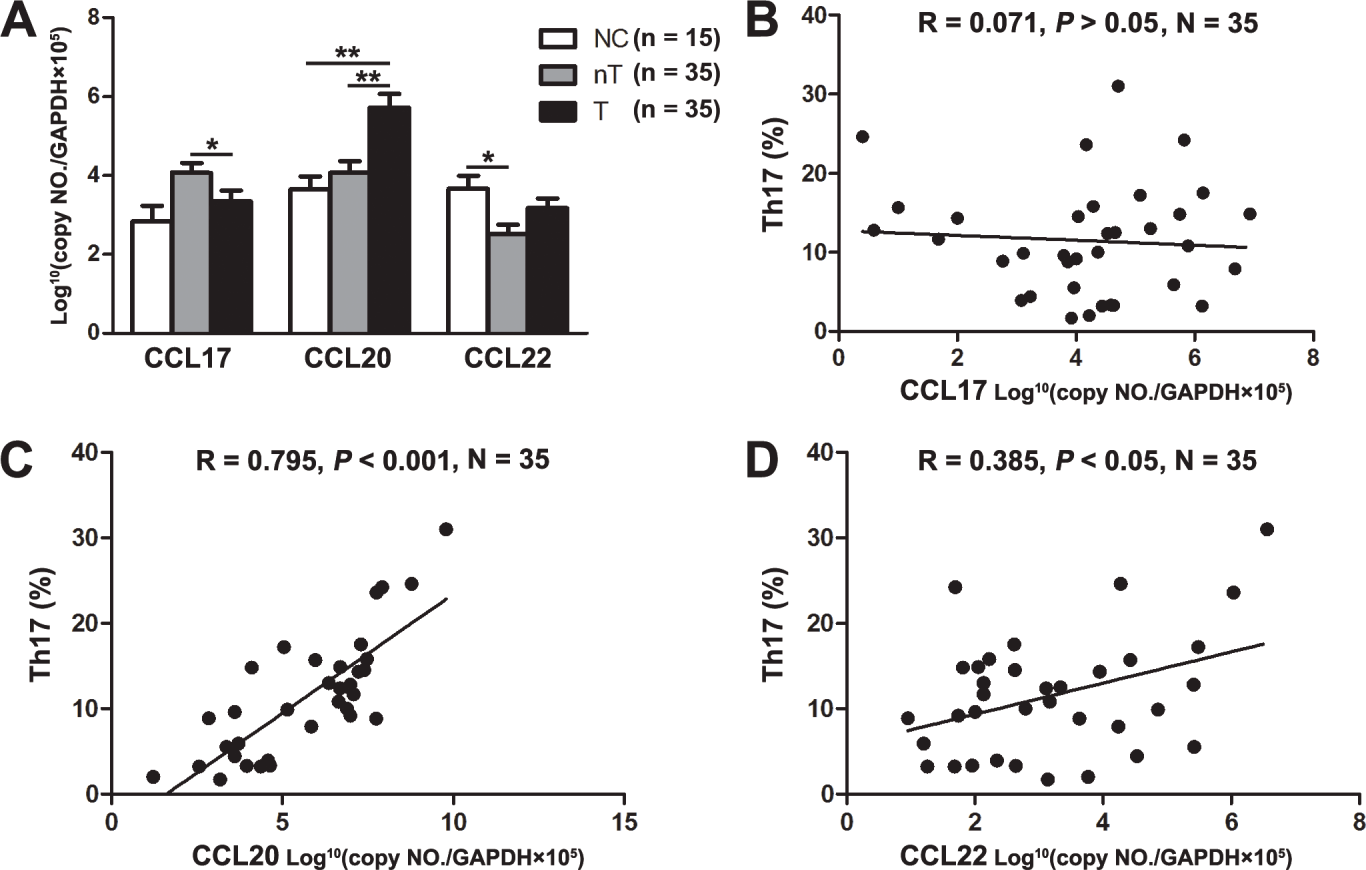
Associations of chemokine levels with intratumoral Th17 cells. (A) Real-time PCR of chemokine CCL17, CCL20 and CCL22 in tumor (T) and non-tumor (nT) regions of patients with cervical cancer and in normal control (NC). The value was normalized to GAPDH, multiplied by 10^5^, and log transformed. *P < 0.05, **P < 0.01. (B, C, D) Correlations of chemokine CCL17 (B), CCL20 (C) and CCL22 (D) with frequency of tumor-infiltrating Th17 cells. Statistical analysis showed a strong correlation of CCL20 with Th17 cells in tumor.

Because Th17 cells highly expressed CCR6 and CCR4, we hypothesized that Th17 cells could migrate in response to CCL20, CCL22 and CCL17. We determined expression of CCL20, CCL22 and CCL17 by real-time PCR. We found significantly higher mRNA expression of CCL20 in the tumor tissues compared to non-tumor tissues and normal control tissues (*P* < 0.01, Fig. 4A). Further, a strongly positive correlation was observed between the prevalence of Th17 cells and intra-tumoral expression of CCL20 (R = 0.795, *P* < 0.001; Fig. 4C). These data suggested that CCL20 is an important signal to mediate the trafficking of Th17 cells to tumors. On the contrary, we did not found increased expression of CCL17 and CCL20, the two specific ligands of CCR4, in tumor tissue (Fig. 4A). Further, regression analysis only found weak correlation of Th17 cells with CCL22 (R = 0.385, *P* < 0.05; Fig. 4D), but not with CCL17 (*P* > 0.05, Fig. 4B), in the same tumor microenvironment. The results collectively indicate that CCR4-CCL17/CCL22 axis may not be mainly responsible for Th17-cell migration.

### Chemokine effects on Th17 cell recruitment

**Fig 5:**
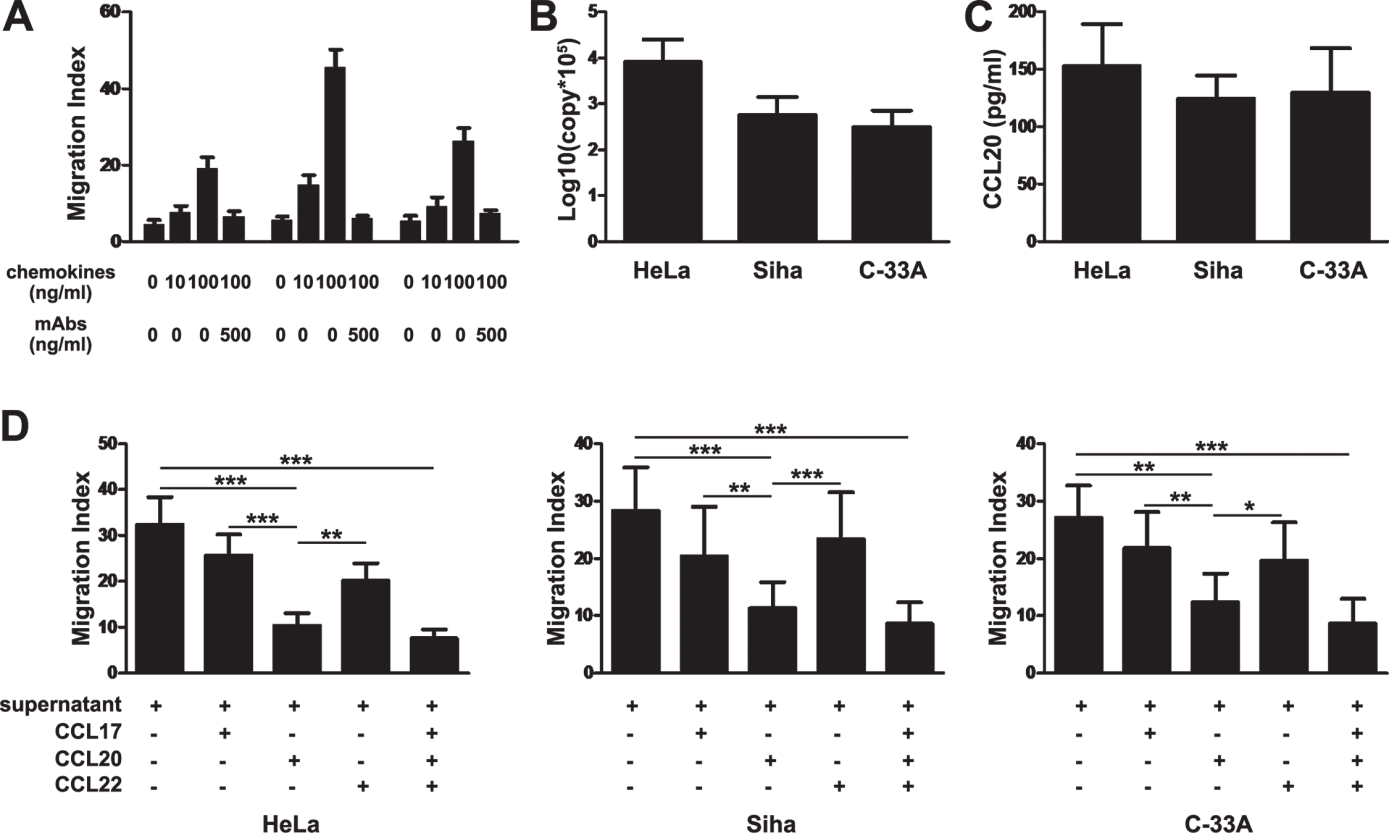
Chemokine effects on Th17 cell recruitment. Migration assays were performed in a Transwell system. (A) Th17 cells migrate in response to recombinant human CCL17, CCL20 and CCL22 in dose-dependent manner (n = 5). Specific antibodies to chemokines significantly inhibit Th17 cell migration.***P < 0.001. (B and C) Expression of CCL20 by HeLa, Siha, and C-33A cells detected using Real-time PRC (B) and ELISA (C). All the three cervical cells could highly secrete CCL20 with highest level of CCL20 by HeLa cells. (D) Th17 cells also migrate toward culture supernatants of HeLa, Siha, and C-33A cells, which can be efficiently blocked by antibody against CCL20 alone or in combination with CCL17 and CCL22, but significantly less effectively by antibody against CCL17 or CCL22 (n = 3). *P < 0.05, **P < 0.01,***P < 0.001.

Significant chemotactic responses of circulating Th17 cells to recombinant human CCL20 were observed in dose-dependent manner (Fig. 5A). Further, a neutralizing monoclonal antibody to CCL20 did markedly blocked CCL20-induced migration of Th17 cells (*P* < 0.001, Fig. 5A). Though CCL17 and CCL22 can also induce migration of Th17 cells, their effect was significantly weaker than CCL20 did (migration index in concentration of 100 ng/ml: CCL20, 45.5 ± 10.6 vs. CCL17, 19.1 ± 6.5 and vs. CCL22, 26.1 ± 7.9, both *P* < 0.001; Fig. 5A). To further determine whether tumor-secreted chemokines had an effect on Th17 cell recruitment, chemotaxis assay using culture supernatants from cervical cell lines was performed. The HeLa cells are HPV-18 infected cervical cells, Siha cells are HPV-16 infected cervical cells, and C-33A cells are HPV negative cervical cancer cells. All the three cervical cells could highly secrete CCL20 with highest level of CCL20 by HeLa cells (Fig. 5B and 5C), and induced significant migration of Th17 cells (Fig. 5D). This can be efficiently blocked by antibody against CCL20 alone or in combination with CCL17 and CCL22 (*P* < 0.001), but significantly less effectively by antibody against CCL17 or CCL22 (Fig. 5B). These results collectively indicate that CCL20 can induce effective migration of Th17 cells into tumor tissues.

## Discussion

As reported in other solid tumors [8, 10, 11, 19, 23–25], the present study showed that substantial Th17 cells, displaying an activated memory phenotype, are concentrated within cervical cancer tissue. However, the role of Th17 cells in cancer immunity remains mixed. Accumulating evidence indicates that although cancer patients exhibit a generalized immunosuppressive status, the inflammatory reaction at tumor site can foster tumor growth and progression. The perpetuation of chronic inflammation is largely achieved through positive feedback loops, which include inflammatory cells producing cytokines that induce chemokine synthesis in malignant and stromal cells leading to prolonged recruitment of inflammatory cells into the tumor microenvironment[26]. Th17 cells are one of the most critical immune cell subsets in this respect and thus have tumor-promoting effect. In patients with hepatocellular carcinoma, colorectal cancer or esophageal carcinoma, high levels of intratumoral Th17 cells were found to be positively associated with poor prognosis[8, 24, 27]. On the other hand, Th17 cells sometimes can provide important help to boost cytotoxic immunity and thus exert tumor-suppressive effects[28, 29]. Our previous study have showed that high intratumoral Th17 cells predict decreased local recurrence in patients with cervical cancer[12], in line with previous reports in ovarian cancer[25] and melanoma[30].

The sources of Th17 cells in cervical cancer tissues may include the trafficking of circulating Th17 cells to tumors and locally induced Th17 cells. The migration of T cells is tightly regulated by chemokine/chemokine receptor interaction[31]. Previous studies showed that recruitment of Th17 cells is governed by multiple pathways, including CCR2-CCL2, CCR4-CCL17/CCL22, and CCR6-CCL20 [15, 16, 19, 32]. In this context, we found the trafficking of circulating Th17 cells into tumor tissue of cervical cancer was preferentially through CCR6-CCL20 pathway. This conclusion is based on two findings. First, CCL20 had markedly elevated expression in tumor tissue and strongly associated with the prevalence of Th17 cells in the same microenvironment. Second, the circulating Th17 from patients with cervical cancer highly expressed CCR6, and selectively migrate in response to CCL20 in vitro. In the past, CCR6 was mainly implicated to be responsible for the inflammatory recruitment of Th17 cells. Fox example, CCR6 is essential for the optimal recruitment of Th17 cells to sites of Th17-mediated inflammation in experimental autoimmune encephalomyelitis (EAE) [7]. However, accumulating recent findings show that the CCR6 on Th17 cells also plays a critical role in tumor development[8, 24]. It is well accepted that the effect of immune cells on tumor development is determined by not only their pro- or anti-potential but also appropriate localization[33]. All these results, together with our data, show Th17 cell-expressed CCR6 is functional and play an important role in the development of cervical cancer. Besides, though CCR4 may also be related to Th17 cell-migration, the effect is much weaker than CCR6 according to less expression of CCR4, lower intratumoral levels of CCL17 and CCL22, and weaker chemotacitc responses to CCL17 and CCL22.

In conclusion, we showed here that CCR6-CCL20 pathway is preferential chemoattractant for the trafficking of circulating Th17 cells into tumor tissue of cervical cancer. The results extend our understanding of the mechanism of cervical carcinogenesis, and provide a potential strategy for treatment of cervical cancer.

## Supporting Information

S1 Table Patients Characteristics. (DOC)
