# Supplementary material for: Preferential Recruitment of Th17 Cells to Cervical Cancer via CCR6-CCL20 Pathway
Source: PLoS One. 2015 Mar 13;10(3):e0120855. doi: 10.1371/journal.pone.0120855 (PMC4359139; doi:10.1371/journal.pone.0120855)
Supplement: S1 Table — (DOC) [file pone.0120855.s001.doc]

**S1 Table.** Patients Characteristics

| Characteristic |  |
| --- | --- |
| Age (range), years | 58.2(38-72) |
| Human papilloma virus (HPV), positive/negative | 29/6 |
| Tumor size (cm), <3.0/≥3.0  Tumor type, squamous/adeno/adenosquamous  FIGO stage, I/II/III  Differentiation, I/II/III  Lymph-vascular space invasion (LVSI), no/yes  Infiltration depth (mm), <15/≥15  Lymph node status (LNS), negative/positive | 13/22  28/5/2  14/13/8  9/11/15  16/19  11/24  17/18 |
